# Reply to the Commentary: Should Injectable Meloxicam Be Approved for Use in Cats?

**DOI:** 10.1111/jvp.70056

**Published:** 2026-03-08

**Authors:** Ludovic Pelligand, Laura Cole, Daniel S. J. Pang

**Affiliations:** ^1^ Department of Clinical Sciences and Services The Royal Veterinary College Hatfield UK; ^2^ Department of Comparative Biological Sciences The Royal Veterinary College Hatfield UK; ^3^ APPRAISE Laboratory (Antimicrobial Pharmacodynamic Pharmacokinetic Research Accelerator and Innovator for Sustainable Endeavours) The Royal Veterinary College Hatfield UK; ^4^ Faculty of Veterinary Medicine University of Calgary Calgary Alberta Canada; ^5^ Faculty of Veterinary Medicine Université de Montréal Saint‐Hyacinthe Québec Canada

**Keywords:** acute kidney injury, adverse drug reactions, anaesthesia, carprofen, NSAIDs, robenacoxib, whole blood assay

We thank Wun et al. (2026) for their comment on the occurrence of peri‐operative Acute Kidney Injury in a clinical trial that was pivotal for the marketing of meloxicam 5 mg/mL solution for cats (U.S. Food and Drug Administration 2004) and subsequent studies (Krekis et al. 2024). We have we been asked to provide a different perspective and we have complemented this letter with PK/PD simulations from available feline NSAID data.

Acute kidney injury (AKI) after surgical procedures in cats has received little attention until recently, although scoping reviews on this topic are emerging in dogs (Quinn 2025). It may therefore be informative to review evidence available from the human literature. In people, the incidence of peri‐operative acute kidney injury is emerging as more common than previously recognised (Meersch et al. 2024). AKI and acute kidney disease (AKD) are a continuum (Chawla et al. 2017) that are described in standardised terms, mainly following the publication of the KDIGO staging (Kidney Disease: Improving Global Outcomes (KDIGO) 2012). Post‐operative AKI (increase in creatinine ≥ 0.3 mg/dL or ≥ 26.5 μmol/L) is an abrupt decrease in kidney function after the initiating anaesthetic/surgical event. It can be transient (recovery within 48 h) or persisting (lasting up to 7 days). After 7 days, the persisting decrease in glomerular filtration rate (GFR) is classified as acute kidney disease (AKD), which may progress to chronic kidney disease (CKD) if persisting beyond 90 days.

The pathophysiology of peri‐operative AKI is complex and likely to involve several predisposing factors, including intra‐operative hypotension, blood loss, peri‐operative systemic inflammation (as a response of the organism to the trauma of surgery) and administration of potentially nephrotoxic drugs (Meersch et al. 2017). Recovery of renal function can be explained through several mechanisms. The first mechanism is an early physiological recovery of normal circulating volume and haemodynamic conditions in the early post‐operative period through resumption of normal water and feed intake. Secondly, there is a possibility of regeneration of lost tubular cells by renal progenitor cells (immature tubular cells with capacity to regenerate entire segments) (Kellum et al. 2021) leading to functional recovery within a few days to weeks. Thirdly, when tubular cells are lost, recovery of renal function is still possible beyond the regeneration stage by compensatory hypertrophy of remaining nephrons, but this happens over several months. The remnant single nephron GFR increase is initially adaptive, but sustained hyperfiltration is ultimately potentially detrimental (maladaptive), increasing the risk of progression to CKD.

What is the prevalence of peri‐operative AKI in cats? Does AKI lead to AKD and predispose cats to CKD? What is the possible contribution of peri‐operative NSAID use in initiating AKI (or exacerbating AKD)? We certainly do not currently have answers to these questions in veterinary medicine but the largest prospective study to date in people brings useful insights. The EPIS‐AKI study was the first prospective international observational multi‐center clinical trial carried out in more than 10,000 patients undergoing major surgical procedures, defined as exceeding a duration of 2 h and requiring subsequent ICU or high dependency unit admission (Zarbock et al. 2023). Changes in serum creatinine were followed daily for the first 3 days following surgery and classified as KDIGO stage 1 if creatinine increased by 0.3 mg/dL (≥ 26.5 μmol/L) within 48 h or 1.5–1.9 times increased from baseline within 72 h after surgery. The prevalence of post‐operative AKI within 72 h after surgery was 18.4% (i.e., 1 in 5 patients). Amongst these, 63.5% were KDIGO stage 1, 25.7% were KDIGO stage 2, and 10.7% were KDIGO stage 3. Of all AKI cases, 76.2% occurred within the first 24 h after surgery and only 34% cases were persistent AKI (duration ≥ 48 h).

A secondary analysis of the same EPIS‐AKI dataset followed the incidence of AKD after 7 days (estimated GFR < 60 mL/min/1.73 m^2^) (Meersch et al. 2024) and included 9510 patients without pre‐existing CKD. Of these, 9.9% of patients developed AKD (decrease in GFR persisting beyond 7 days). AKI was a key driver for AKD: 14.9% of patients with KDIGO Stage 1 developed AKD after 7 days and AKD was twice *less* likely if AKI was transient. In the multivariate analysis, aminoglycosides were independently associated with the development of AKD but peri‐operative NSAIDs appeared rather protective against AKD (OR 0.78, 95% CI 0.64–0.94). The authors recognised that this counterintuitive result could possibly be due to unexplained confounding factors and selection bias; however, there was no evidence based on the data to consider NSAIDs as a contributing factor of AKD within the context of this study population. EPIS‐AKI could inform a larger‐scale study in hospitalised cats but the population of cats undergoing spay would arguably be younger, healthier and exposed to shorter surgeries. We therefore urge for a prospective multicentric controlled clinical trial with appropriate postoperative follow‐up (ethics of sampling and diagnostic capability) to estimate the prevalence of AKD in cats following sterilisation after receiving (or not) an NSAID at the discretion of the clinician.

It is possible that NSAIDs, presumably through inhibition of cyclooxygenase (COX)‐1, facilitate a reduction in glomerular filtration rate (GFR) in response to anaesthesia by inhibiting tubuloglomerular feedback, although this is not necessarily associated with acute tubular damage in a euvolaemic cat. Water deprivation in experimental mice show an upregulation of COX‐2 in the medulla of the kidney (Küper et al. 2011). There, COX‐2 seems required to allow the interstitial cells to survive the hypertonic stress resulting from the increased osmolality generated in the interstitium in response to water deprivation (Pelligand and Elliott 2017). Indeed, COX‐2 activity is linked to the ability of these cells to generate organic osmolytes intracellularly to compensate osmotic stress in the short term, whilst the cells adapt to hypertonicity, which requires some time. If COX‐2 is inhibited at the same time as water deprivation occurs, the interstitial cells undergo apoptosis (programmed cell death mediated by caspase‐3), resulting in death of medullary cells. The protective role of COX‐2 for medullary cells in water deprivation situations has not been investigated in cats but the distribution of COX enzymes within the medulla of the feline kidney suggests the same regulation could occur in cats (Pelligand et al. 2015). If inadequate post‐operative pain relief could result in the cats not eating or drinking properly in the immediate post‐operative period (with increased osmotic stress as a promoter of early AKI), the risk of withholding NSAID analgesia should therefore also be considered. Wun et al.'s argument would be that any increase in BUN/creatinine is relevant if it is an avoidable problem (such as NSAID‐induced) and they question whether NSAIDs still have an essential role in peri‐operative analgesia. With better understanding of opioid pharmacology, implementation of multimodal analgesia and development of regional analgesia techniques, and improved physiological monitoring compared to 20 years ago in 2005, perhaps adequate analgesia could be achieved without the use of NSAIDs peri‐operatively in some cases. However, it depends on the clinical setting and both arguments can be made, that is, appropriate use of pre−/intra‐operative NSAIDs versus only post‐operative use.

Until now the question of selectivity of COX inhibition has not been considered in the debate. From the original PK data, the lead author (LP) has modelled the median peri‐operative concentrations of plasma meloxicam, plasma carprofen and blood robenacoxib after a single subcutaneous administration of 0.2, 4 (2 mg/kg of the enantiomer S(+)) and 2 mg/kg, the respective licensed doses (Taylor et al. 1996; Giraudel et al. 2005; Pelligand et al. 2016) (Figure 1). The corresponding plasma (haematocrit adjusted) and blood concentrations for COX‐1 and COX‐2 (converted to μg/L) were sourced from feline Whole Blood Assays studies (Giraudel et al. 2005, 2009). The inhibition ratio IC_50_COX‐1/IC_50_COX‐2 for meloxicam, S(+) carprofen and robenacoxib are 3.5, 28.1 and 502.3. If the purpose of index comparison is to estimate margin of clinical safety, the ratio of IC_20_ COX‐1 (> 20% inhibition could affect homeostasis including regulation of renal perfusion) to IC_80_ COX‐2 (> 80% inhibition predicts anti‐inflammatory/analgesia efficacy) should be considered (Warner et al. 1999; Lees et al. 2004).

**FIGURE 1:**
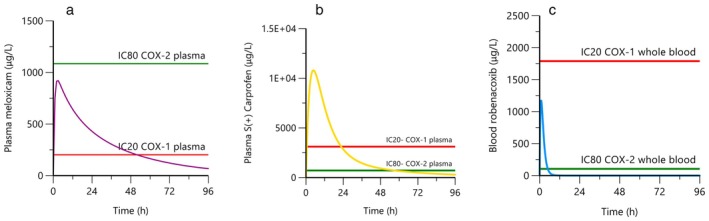
Median concentrations of plasma meloxicam (purple, a), plasma carprofen (yellow gold, b) and blood robenacoxib (blue, c) after a single subcutaneous administration of 0.2, 4 (2 mg/kg of the enantiomer S(+)) and 2 mg/kg, respectively. Relevant computed IC_20_COX‐1 and IC_80_COX‐2 are represented by a red and a green horizonal line, respectively.

After a single subcutaneous injection of meloxicam at 0.2 mg/kg, data predict a long lasting (> 48 h) inhibition of COX‐1 above 20% for a modest inhibition of COX‐2 (Figure 1a). After a single subcutaneous injection of carprofen at 4 mg/kg (2 mg/kg of the active S(+) enantiomer, Figure 1b), COX‐1 inhibition above 20% lasts for 24 h, whereas COX‐2 inhibition above 80% lasts for up to 48 h. After a single subcutaneous injection of robenacoxib at 2 mg/kg (Figure 1c), there is a short‐lasting inhibition of COX‐2 above 80%, whereas COX‐1 is untouched.

This is not to say that dosage regimen should be solely determined based on in vitro whole blood assays, as the analgesia benefit is driven by tissue concentrations and the risk/benefit balance could also depend on NSAIDs effects not mediated by COX. For example, ex vivo feline data confirm inhibition of Thromboxane B_2_ formation with licensed doses of meloxicam in the cat (0.3 mg/kg sc in Schmid et al. 2010, 0.2 mg/kg sc in Krekis et al. 2024). We wanted to illustrate the question of selectivity at licensed doses.

In conclusion, a non‐azotaemic increase in creatinine (IRIS AKI Grade I) does not necessarily indicate intrinsic kidney injury. Serial increases in serum creatinine should be interpreted alongside other diagnostic tests, including urine analysis and fractional excretion of electrolytes to help differentiate fluid responsive AKI versus intrinsic AKI (Troìa et al. 2018). This differentiation may be made through urine analysis to assess for evidence of tubular injury (renal glucosuria with normoglycaemia, proteinuria with an inactive sediment and urinary casts). Monitoring serum creatinine in response to intravenous fluid therapy can also help distinguish real tubular injury as the origin of the change in creatinine versus a post‐operative haemoconcentration and a fluid responsive AKI (Segev et al. 2024). We need to develop renal recovery clinics (https://www.rvc.ac.uk/research/projects/acute‐kidney‐injury‐renal‐recovery‐clinic) for tracking trajectories of renal function after AKI, validate the utility of renal tubular injury biomarkers for early detection of AKI and to assess their role in guiding early therapeutic interventions in veterinary nephrology. This knowledge is required before a well‐informed risk–benefit analysis can be used to determine whether changes to current clinical practice are needed.

## Author Contributions

**L.P.:** conceptualization, formal analysis, software, visualisation, writing – original draft preparation, writing – review and editing. **L.C.:** investigation, writing – original draft preparation, writing – review and editing. **D.S.J.P.:** conceptualization, writing – original draft preparation, writing – review and editing.

## Disclosure

L. Pelligand received a CASE award from the BBSRC and Novartis Animal Health for his PhD, from 2006 to 2010.

## Conflicts of Interest

The authors declare no conflicts of interest.

## Data Availability

Data sharing not applicable to this article as no datasets were generated or analysed during the current study.

## References

[jvp70056-bib-0001] Chawla, L. S. , R. Bellomo , A. Bihorac , et al. 2017. “Acute Kidney Disease and Renal Recovery: Consensus Report of the Acute Disease Quality Initiative (ADQI) 16 Workgroup.” Nature Reviews. Nephrology 13, no. 4: 241–257. 28239173 10.1038/nrneph.2017.2

[jvp70056-bib-0002] Giraudel, J. M. , A. Diquelou , V. Laroute , P. Lees , and P. L. Toutain . 2005. “Pharmacokinetic/Pharmacodynamic Modelling of NSAIDs in a Model of Reversible Inflammation in the Cat.” British Journal of Pharmacology 146, no. 5: 642–653. 16113689 10.1038/sj.bjp.0706372 PMC1751204

[jvp70056-bib-0003] Giraudel, J. M. , P. L. Toutain , J. N. King , and P. Lees . 2009. “Differential Inhibition of Cyclooxygenase Isoenzymes in the Cat by the NSAID Robenacoxib.” Journal of Veterinary Pharmacology and Therapeutics 32, no. 1: 31–40. 19161453 10.1111/j.1365-2885.2008.01031.x

[jvp70056-bib-0005] Kellum, J. A. , P. Romagnani , G. Ashuntantang , C. Ronco , A. Zarbock , and H.‐J. Anders . 2021. “Acute Kidney Injury.” Nature Reviews Disease Primers 7, no. 1: 52. 10.1038/s41572-021-00284-z 34267223

[jvp70056-bib-0006] Kidney Disease: Improving Global Outcomes (KDIGO) . 2012. “Acute Kidney Injury Work Group. KDIGO Clinical Practice Guideline for Acute Kidney Injury.” Kidney International 2: 1–138.

[jvp70056-bib-0007] Krekis, A. , J. N. King , D. D'Arcy‐Howard , N. Stapleton , J. Elliott , and L. Pelligand . 2024. “Effect of Meloxicam or Robenacoxib Administration Timing on Renal Function and Postoperative Analgesia in Cats Undergoing Ovariohysterectomy: A Randomized, Blinded, Controlled Clinical Trial.” Journal of Veterinary Pharmacology and Therapeutics 47, no. 3: 175–186. 38235901 10.1111/jvp.13427

[jvp70056-bib-0008] Küper, C. , H. Bartels , F.‐X. Beck , and W. Neuhofer . 2011. “Cyclooxygenase‐2‐Dependent Phosphorylation of the Pro‐Apoptotic Protein Bad Inhibits Tonicity‐Induced Apoptosis in Renal Medullary Cells.” Kidney International 80, no. 9: 938–945. 21716255 10.1038/ki.2011.199

[jvp70056-bib-0009] Lees, P. , M. F. Landoni , J. Giraudel , and P. L. Toutain . 2004. “Pharmacodynamics and Pharmacokinetics of Nonsteroidal Anti‐Inflammatory Drugs in Species of Veterinary Interest.” Journal of Veterinary Pharmacology and Therapeutics 27, no. 6: 479–490. 15601442 10.1111/j.1365-2885.2004.00617.x

[jvp70056-bib-0010] Meersch, M. , C. Schmidt , and A. Zarbock . 2017. “Perioperative Acute Kidney Injury: An Under‐Recognized Problem.” Anesthesia and Analgesia 125, no. 4: 1223–1232. 28787339 10.1213/ANE.0000000000002369

[jvp70056-bib-0011] Meersch, M. , R. Weiss , C. Strauß , et al. 2024. “Acute Kidney Disease Beyond Day 7 After Major Surgery: A Secondary Analysis of the EPIS‐AKI Trial.” Intensive Care Medicine 50, no. 2: 247–257. 38285051 10.1007/s00134-023-07314-2 PMC10907445

[jvp70056-bib-0012] Pelligand, L. , and J. Elliott . 2017. “Effects of Non‐Steroidal Anti‐Inflammatory Drug Treatment on the Kidney.” In BSAVA Manual of Canine and Feline Nephrology and Urology. British Small Animal Veterinary Association.

[jvp70056-bib-0013] Pelligand, L. , A. Soubret , J. N. King , J. Elliott , and J. P. Mochel . 2016. “Modeling of Large Pharmacokinetic Data Using Nonlinear Mixed‐Effects: A Paradigm Shift in Veterinary Pharmacology. A Case Study With Robenacoxib in Cats.” CPT: Pharmacometrics & Systems Pharmacology 5, no. 11: 625–635. 27770596 10.1002/psp4.12141 PMC5193001

[jvp70056-bib-0014] Pelligand, L. , N. Suemanotham , J. N. King , et al. 2015. “Effect of Cyclooxygenase (COX)‐1 and COX‐2 Inhibition on Furosemide‐Induced Renal Responses and Isoform Immunolocalization in the Healthy Cat Kidney.” BMC Veterinary Research 11: 296. 26634699 10.1186/s12917-015-0598-z PMC4669647

[jvp70056-bib-0015] Quinn, C. T. 2025. “Time Course of Clinical Signs and Mortality in Dogs With Severe Perioperative Acute Kidney Injury: A Scoping Review.” Australian Veterinary Journal 103, no. 7: 443–449. 40421852 10.1111/avj.13454 PMC12213321

[jvp70056-bib-0016] Schmid, V. B. , W. Seewald , P. Lees , and J. N. King . 2010. “In Vitro and Ex Vivo Inhibition of COX Isoforms by Robenacoxib in the Cat: A Comparative Study.” Journal of Veterinary Pharmacology and Therapeutics 33, no. 5: 444–452. 20840388 10.1111/j.1365-2885.2010.01166.x

[jvp70056-bib-0017] Segev, G. , S. Cortellini , J. D. Foster , et al. 2024. “International Renal Interest Society Best Practice Consensus Guidelines for the Diagnosis and Management of Acute Kidney Injury in Cats and Dogs.” Veterinary Journal 305: 106068. 38325516 10.1016/j.tvjl.2024.106068

[jvp70056-bib-0018] Taylor, P. M. , P. Delatour , F. M. Landoni , et al. 1996. “Pharmacodynamics and Enantioselective Pharmacokinetics of Carprofen in the Cat.” Research in Veterinary Science 60, no. 2: 144–151. 8685536 10.1016/s0034-5288(96)90009-0

[jvp70056-bib-0019] Troìa, R. , M. Gruarin , C. Grisetti , et al. 2018. “Fractional Excretion of Electrolytes in Volume‐Responsive and Intrinsic Acute Kidney Injury in Dogs: Diagnostic and Prognostic Implications.” Journal of Veterinary Internal Medicine 32, no. 4: 1372–1382. 29770972 10.1111/jvim.15146 PMC6060310

[jvp70056-bib-0020] U.S. Food and Drug Administration . 2004. Supplemental NADA 141–219, Metacam, Meloxicam 5 mg/mL Solution for Injection. U.S. Food and Drug Administration.

[jvp70056-bib-0021] Warner, T. D. , F. Giuliano , I. Vojnovic , A. Bukasa , J. A. Mitchell , and J. R. Vane . 1999. “Nonsteroid Drug Selectivities for Cyclo‐Oxygenase‐1 Rather Than Cyclo‐Oxygenase‐2 Are Associated With Human Gastrointestinal Toxicity: A Full In Vitro Analysis.” Proceedings of the National Academy of Sciences of the United States of America 96, no. 13: 7563–7568. 10377455 10.1073/pnas.96.13.7563 PMC22126

[jvp70056-bib-0023] Wun, M. K. , M. H. Court , N. F. Villarino , and R. Malik . 2026. “Should Injectable Meloxicam be Approved for Use in Cats?” Journal of Veterinary Pharmacology and Therapeutics 49, no. 2: 228–230.10.1111/jvp.7006041797288

[jvp70056-bib-0022] Zarbock, A. , R. Weiss , F. Albert , et al. 2023. “Epidemiology of Surgery Associated Acute Kidney Injury (EPIS‐AKI): A Prospective International Observational Multi‐Center Clinical Study.” Intensive Care Medicine 49, no. 12: 1441–1455. 37505258 10.1007/s00134-023-07169-7 PMC10709241

